# Targeting clock-controlled gene *Nrf2* ameliorates inflammation-induced intervertebral disc degeneration

**DOI:** 10.1186/s13075-022-02876-w

**Published:** 2022-08-03

**Authors:** Pandi Peng, Dong Wang, Xiaolong Xu, Di Wang, Bo Gao, Han Wang, Haoruo Jia, Qiliang Shang, Chao Zheng, Chu Gao, Jianxin Mao, Zhuojing Luo, Liu Yang, Xueyu Hu

**Affiliations:** 1 Institute of Orthopedic Surgery, Xijing Hospital, Fourth Military Medical University, Xi’an, 710032 People’s Republic of China; 2 Medical Research Institute, Northwestern Polytechnical University, Xi’an, 710072 People’s Republic of China

**Keywords:** Intervertebral disc degeneration, Circadian rhythms, Bmal1, NRF2, Inflammation

## Abstract

**Background:**

Intervertebral disc (IVD) is a highly rhythmic tissue, which experiences a diurnal cycle of high/low mechanical loading via the changes of activity/rest phase. There are signs that disruption of the peripheral IVD clock is related to the process of intervertebral disc degeneration (IDD). However, it is still unclear whether inflammation could disturb the IVD clock and thus induce the process of IDD.

**Methods and results:**

In this study, we used IL-1β, a commonly used inflammatory factor, to induce IDD and found that the IVD clock was dampened in degenerated human nucleus pulposus specimens, rat nucleus pulposus (NP) tissues, and cells. In this study, we found that the circadian clock of NP cells was totally disrupted by knockdown of the core clock gene brain and muscle arnt-like protein-1 (*Bmal1*), which thus induced the dysfunction of NP cells. Next, we explored the mechanism of dampened clock-induced IDD and found that knockdown of *Bmal1* decreased the expression of nuclear factor erythroid2-related factor 2 (NRF2), a downstream target gene of *Bmal1*, and increased inflammatory response, oxidative stress reaction, and apoptosis of NP cells. In addition, NRF2 activation attenuated the dysfunction of NP cells induced by the dampened IVD clock and the degenerative process of NP tissues in an organotypic tissue-explant model.

**Conclusions:**

Taken together, our study extends the relationship between peripheral clock and IVD homeostasis and provides a potential therapeutic method for the prevention and recovery of IDD by targeting the clock-controlled gene *Nrf2*.

**Supplementary Information:**

The online version contains supplementary material available at 10.1186/s13075-022-02876-w.

## Introduction

Intervertebral disc degeneration (IDD) is the major cause of low back pain, a common musculoskeletal disorder [1, 2]. As the population aged, the morbidity and disability rate of IDD are increasing, which imposes a negative effect on the work and life of patients and places an insupportable burden on the society [3–5]. Intervertebral discs (IVDs) are located between adjacent vertebral bodies and consist of three independent parts, including the inner nucleus pulposus (NP), the external annulus fibrosus, and the upper and lower cartilage endplates [6]. NP tissue is the critical component of IVD, which maintains the shape and function of the IVD by regulating the dynamic balance of the extracellular matrix (ECM). NP dysfunction plays a key role in the pathological process of IDD. At present, it is believed that the occurrence and development of IDD are affected by aging, abnormal stress, inflammation, and so on [7, 8], but the molecular mechanism is still unclear.

Circadian rhythm is a widespread phenomenon in the natural world [9]. The circadian clock of the mammal drives ∼24-hour diurnal rhythms in physiology and behavior to adapt to the expected changes of the day-night environment, which plays a pivotal role in maintaining the physical homeostasis [10, 11]. The production of a circadian rhythm depends on the coordinated expression of a series of clock genes, including *Bmal1* (brain and muscle Arnt-like protein-1), *Clock* (circadian locomotor output cycles kaput), *Per1/2* (Period), *Cry1/2* (Cryptochrome), *Rorα/β/δ/γ* (retinoic acid-related orphan receptor), and *Rev-ERbα/β* (nuclear receptor subfamily 1, group D), which forms a transcription–translation feedback loop [12–15].

BMAL1 is a core clock transcription factor, which promotes the transcription of downstream clock-controlled genes (CCGs) by targeting the E-box structure in their promoter region to regulate the physiological functions of the body. Studies showed that the catabolism of human articular chondrocytes was triggered when *Bmal1* was knocked down by siRNA [16–18]. In addition, *Bmal1*-specific ablation in mouse chondrocytes (*Col2α1-Bmal1*^*−/−*^) caused progressive degeneration of articular cartilage [16]. These studies fully demonstrated that the clock molecule BMAL1 plays an important role in maintaining the homeostasis of ECM in articular cartilage. Although some phenotypes of IDD were reported in BMAL1-deficient mice, the potential role of BMAL1 in regulating IVD homeostasis remains unclear [10, 19].

Inflammation is one of the main risk factors of IDD. It was reported that inflammation significantly destroyed the intrinsic rhythm of some peripheral tissues, such as kidney, liver, heart, and so on [20–22], suggesting that inflammation in IVD microenvironment might induce the process of IDD by disrupting the peripheral rhythm. Nuclear factor erythroid2-related factor 2 (NRF2), a classic player in redox control and inflammatory regulation, was reported to be directly controlled by the circadian transcription factor BMAL1 in lung and pancreatic tissue [23, 24]. However, it is still unclear whether disruption of NRF2 expression mediates the IDD induced by dampened IVD rhythm.

In this study, we found the expression of BMAL1 was reduced in degenerated human NP tissue, rat NP cells, and tissues. And we also found that knockdown of *Bmal1* decreased the expression of NRF2 and increased inflammatory response, oxidative stress reaction, and apoptosis of NP cells in vitro. Sulforaphane (SFN), an agonist of *Nrf2*, attenuated si-*Bmal1*-induced disfunction of NP cells in vitro and the degeneration of rat NP tissues in organotypic tissue-explant culture. Our study provided a new understanding of the pathogenesis of IDD and put forward a therapeutic strategy for the prevention and treatment of IDD.

## Materials and methods

### Reagents and antibodies

IL-1β (Pepro Tech, USA), Forskolin (APExBIO Technology, USA), SFN (APExBIO Technology, USA), Rat IL-1β ELISA Kit (Elabscience, China), Rat TNF-α ELISA Kit (Elabscience, China). For immunohistochemical and immunofluorescence analyses, the following antibodies were used: anti-BMAL1 (Abcam, #ab3350), anti-NRF2 (Proteintech, #16396-1-AP), anti-Aggrecan (Proteintech, #13880-1-AP), anti-MMP13 (Abcam, #ab39012), anti-p65 (Santa Cruz Biotechnology, #(F-6): sc-8008), anti-Phospho-p65(Santa Cruz Biotechnology, #(A-8): sc-166748), and GAPDH (Proteintech, #60004-1-lg). For immunohistochemical and immunofluorescence analyses, the following antibodies were used: anti-BMAL1 (Abcam, #ab3350), anti-NRF2 (Proteintech, #16396-1-AP), anti-Aggrecan (Proteintech, #13880-1-AP), anti-MMP13 (Abcam, #ab39012), and anti-p65(Invitrogen, PA5-16545).

### Donor samples

Human NP specimens were obtained from 14 donors (6 males and 8 females; mean age = 48.8 ± 11.1 years), undergoing disc surgery for the treatment of disc herniation, IVD degeneration, or scoliosis, in accordance with the approval of the Medical Ethics Committee of the First Affiliated Hospital of the Air Force Military Medical University (KY20203146-1). NP specimens were graded according to the modified Pfirrmann grading system by magnetic resonance imaging (MRI) [25]. The MRI images and slides were blinded to the observers and randomized using a random number method. The degree of IDD was assessed by 3 other blinded orthopedic surgeons (Xiaolong Xu, Han Wang and Haoruo Jia) according to modified Pfirrmann grading system by MRI. According to the Pfirrmann grade, NP specimens were divided into two groups: the mildly degenerated group, which contains grade II (*n*=3) and grade III (*n*=4) samples; the severely degenerated group, which contains grade IV (*n*=3) and grade V (*n*=4). The demographic data of donors was shown in Table 1.

**Table 1:** Demographic data of donors

Donor no.	Age	Gender	Level	Pfirrmann grading
**mildly degenerated**				
	**40**	**Male**	**L5/S1**	**II**
	**29**	**Female**	**L5/S1**	**II**
	**65**	**Female**	**L3/L4**	**II**
	**47**	**Male**	**L2/L3**	**III**
	**56**	**Female**	**L5/S1**	**III**
	**46**	**Female**	**L5/S1**	**III**
	**63**	**Female**	**L5/S1**	**III**
**severely degenerated**				
	**58**	**Male**	**L4/L5**	**IV**
	**30**	**Male**	**L4/L5**	**IV**
	**52**	**Female**	**L4/L5**	**IV**
	**54**	**Male**	**L5/S1**	**V**
	**57**	**Male**	**L4/L5**	**V**
	**44**	**Female**	**L4/L5**	**V**
	**42**	**Female**	**L5/S1**	**V**

### Cell culture and treatment

The rat cell line was a gift from Prof. Di Chen (Shenzhen Institute of Advanced Technology, Chinese Academy of Sciences). Cells were isolated from the NP of lumbar discs of a single 2-month-old adult Sprague Dawley rat weighing between 250 and 300g. As reported by Chen Di et al. [26], The cells were treated with 0.2% pronase and 0.025% collagenase P overnight for digestion and cultured with Dulbecco’s modified Eagle’s medium (DMEM)/F-12 (1:1) (Gibco, USA) supplemented with 20% (v/v) fetal bovine serum (FBS) (Gibco, USA) and antibiotics. From day 3, cells were grown in the presence of 10 μM Y-27632 and subcultured by trypsinization. It has been confirmed that the level of rTERT mRNA increased with the passaging of NP cells in the presence of Y-27632, which means that the cells are immortalized cells [26]. And the passage numbers of the cell line are 5-10. Rat NP cell line was cultured in DMEM/F-12 (1:1) (Gibco, USA) supplemented with 20% (v/v) FBS (Gibco, USA), penicillin (100 units/mL), and streptomycin (100 μg/mL) under standard conditions (37°C, 21% O_2_, 5% CO_2_).

### siRNA transfection

The siRNA targeting *Bmal1* of rat and negative control siRNA were synthesized by Genepharma (Shanghai, China). The sequences of siRNA were shown in Supplementary Table 1. Rat NP cells were transfected with siRNA duplexes targeting *Bmal1* by Lipofectamine 2000 Reagent (Invitrogen, USA) according to the manufacturer’s instructions. Briefly, Rat NP cells were seeded (2 × 10^4^ cells/mL) in a six-well plate at 24 h before transfected, and the medium of cells was replaced by Opti-MEM (Gibco, USA, #31985070) at 2 h before transfected. Then adding *Bmal1* siRNA or NC siRNA to Opti-MEM was to transfect rat NP cells under the concentration of 80 nM by Lipofectamine 2000 Reagent at 37°C. And Opti-MEM was removed and replaced with DMEM/F-12 containing 20% FBS, 1% penicillin and 1% streptomycin. The knockdown efficacies were verified by quantitative real-time PCR (qRT-PCR) 24 h after transfection and Western blotting 48 h after transfection.

### RNA extraction and qRT-PCR analysis

Rat NP cells were synchronized by 10 μM forskolin for 1 h. Zeitgeber time (ZT) refers to environmental variables that are capable of acting as circadian time cues. Under the formalism of ZT, each full cycle is divided into 24 equal “hours.” Twenty-four hours after synchronization ends, the time was defined as ZT0. NP cells were collected at ZT0, ZT4, ZT8, ZT12, ZT16, ZT20, and ZT24. Total RNA of rat NP cells was isolated using the Total RNA Kit (Omega Biotek, Norcross, GA, USA) according to the manufacturer’s instructions. The RNA concentrations of NP cells were measured by a microplate reader (BioTek, USA) using the ratio of the absorbance at 260 nm to that at 280 nm. And total RNA was converted to cDNA with PrimeScript™ RT Master Mix (TaKaRa, Tokyo, Japan). Then the cDNA product was subjected to qRT-PCR on a 7500 Real-Time PCR System (Applied Biosystems, Foster City, CA, USA) with TB Green Premix Ex Taq II (TaKaRa, Tokyo, Japan). Target gene expression levels were normalized to GAPDH, and relative fold changes in expression were calculated by the 2^-(ΔΔCt)^ method. The primers used for qRT-PCR were listed in Supplementary Table 2.

### Protein extraction and Western blotting analysis

NP cells were washed with phosphate-buffered saline (PBS) twice. Then the cells were lysed in radioimmune precipitation assay (RIPA) buffer (Beyotime Biotech, Nantong, China) with a complete protease inhibitor cocktail (Roche, Germany). The total protein of NP cells was collected by centrifuging at 13,000 rpm for 15 min at 4°C. The concentrations of protein were measured using Pierce BCA Protein Assay Kit (Thermo Fisher Scientific, USA). The total proteins were diluted by loading buffer, heated at 100°C for 15 min, and then certain amounts of protein were separated in 10% sodium dodecyl sulfate-polyacrylamide gels (SDS-PAGE) and transferred to nitrocellulose membranes (Millipore). Then the nitrocellulose membranes were blocked by 5% skim milk in Tris-buffered saline containing 0.1% Tween 20 (TBST) for 40 min at room temperature.

The membranes were incubated overnight at 4°C with primary antibodies, including the following: anti-BMAL1 (diluted 1:1000), anti-NRF2 (diluted 1:1000), anti-Aggrecan (diluted 1:1000), anti-MMP13 (diluted 1:1000), anti-p65 (diluted 1:1000), anti-phospho-p65 (diluted 1:1000), and anti-glyceraldehyde 3-phosphate dehydrogenase (GAPDH) (diluted 1:2000). Then the membranes were incubated with the horseradish peroxidase (HRP)-linked goat anti-rabbit IgG or horse anti-mouse IgG secondary antibody for 1h at a 1:2000 dilution at room temperature. The nitrocellulose membranes were washed three times with TBST for 15 min after each step. Finally, the nitrocellulose membranes were visualized by Immobilon Western Chemiluminescent HRP Substrate (Millipore Corporation, Germany, #WBKLS0100), and the density of nitrocellulose membranes was quantified by Image J software (National Institutes of Health, Bethesda, MD, USA).

### Organotypic tissue-explant of IVD

Under the approval of the Animal Experiment Administration Committee of the Air Force Military Medical University, tails were collected from 10 8-week Sprague-Dawley rats under pathogen-free conditions, after they were euthanized. The muscles and tendons were dissected and removed by a scalpel and surgical scissors under aseptic conditions. Explants consist of the intervertebral disc and two adjacent vertebrae. The explants were randomly divided into 4 groups: uninjured group (control), injured group (needle-punctured), injured and low doses of SFN-treated group (Needle-punctured + 5 μM SFN), and injured and high doses of SFN-treated group (Needle-punctured + 10 μM SFN). Annulus fibrosus (AF) was stabbed with a 20 G needle to enter the center of the NP for 1 min. The explants of Control and Needle-punctured groups were cultured in DMEM/F-12 (1:1) and the explants of Needle-punctured + 5μM SFN and Needle-punctured + 10μM groups were cultured in DMEM/F-12 (1:1) with certain amounts of SFN. The medium was replaced with fresh medium every other day. After 0 days, the explants of Control and Needle-punctured groups were collected and after 0 days, 7 days, and 14 days, the explants of all groups were collected. No explant was excluded from the analysis.

### Histology, immunohistochemistry, and immunofluorescence staining

Human NP specimens were fixed in 4% paraformaldehyde for 48 h, dehydrated in gradually increasing concentrations of sucrose solution, and embedded in the optimal cutting temperature compound (OCT). The IVD of rats were fixed in 4% paraformaldehyde for 48 h, decalcified in 10% ethylenediaminetetraacetic acid (EDTA; pH 7.4) for 14 days, dehydrated in gradually increasing concentrations of sucrose solution, and embedded in OCT. Human NP specimens and coronal-oriented sections of rat IVD tissues were cut to 7 μm. The sections were stained with hematoxylin-eosin (HE) and safranin O-fast green (SO) according to standard protocols. And the modified grading method, which was listed in Supplementary Table 3, was used to evaluate histological scores [4, 27].

Immunohistochemistry (IHC) and immunofluorescence (IF) staining were performed according to standard protocols. The sections of human NP tissue and explants of rat IVD were incubated in 0.2 mg/mL pepsin for 10 min at 37°C for antigen retrieval and then blocked in blocking solution (QuickBlock™, Beyotime Biotech) for 1 h at room temperature. Rat NP cells were fixed in 4% paraformaldehyde for 15 min at room temperature and permeabilized in 0.3% Triton X-100. The sections and cells were then incubated overnight at 4 °C with primary antibodies, including the following: anti-BMAL1 (diluted 1:200), anti-NRF2 (diluted 1:200), anti-AGGRECAN (diluted 1:200), anti-MMP13 (diluted 1:200), or anti-p65(diluted 1:200). Then they were washed with PBS for 5 min three times. Horseradish peroxidase-streptavidin detection system (ZSGB-BIO, Beijing, China) was used for immunohistochemical staining. For immunofluorescence assays, the sections and cells were incubated with fluorescein isothiocyanate (FITC)-conjugated secondary antibodies or Cy3-conjugated secondary antibodies (Proteintech, China). After three washings with PBS, they were incubated in 5 μg/mL 4′,6-diamidino-2-phenylindole (DAPI) for 1 h at room temperature in the dark. Images of the sections and cells were analyzed under a fluorescence microscope (BX53, OLYMPUS, Japan) and fluorescence signals were quantified with Image J software.

### TUNEL assay

TUNEL assay was performed using the One Step TUNEL Apoptosis Assay Kit (Beyotime Biotech), according to the manufacturer’s instructions. In brief, cells were fixed in 4% paraformaldehyde for 15min at room temperature and permeabilized in 0.3% Triton X-100 for 15 min at room temperature and rinsed with 50 μL of TUNEL reaction mixture for 1h at 37°C in the dark. Next, the cells were incubated with DAPI for 10 min. Images were analyzed under a fluorescence microscope (BX53, OLYMPUS, Japan). Total cells and positive cells were quantified with Image J software.

### Reactive oxygen species detection

Reactive oxygen species (ROS) were detected with the oxidation-sensitive fluorescent dye, 2′,7′-dichlorofluorescein diacetate (DCFDA) (Beyotime Biotech). NP cells were washed with PBS twice and stained with 10 μM DCFDA in Opti-MEM for 30 min in the dark at 37°C. Images were analyzed under a fluorescence microscope (Axio Imager A1; Carl Zeiss) and fluorescence signals were quantified with Image J software.

### Statistical analysis

The data were presented as means ± standard error of the mean (SEM) from at least three independent experiments. Whether the data present a normal distribution was tested by the Shapiro Wilk test or D’Agostino test. The differences of two or multiple groups were analyzed by Student’s *t*-test or one-way analysis of variance followed by Tukey’s multiple-comparison post hoc test, respectively. All statistical analyses were performed with SPSS 22.0 and GraphPad Prism 7.0 software. Differences were considered statistically significant at P-value <0.05.

## Results

### IVD clock was dampened in degenerated human nucleus pulposus specimens, rat nucleus pulposus tissues, and cells.

**Fig. 1:**
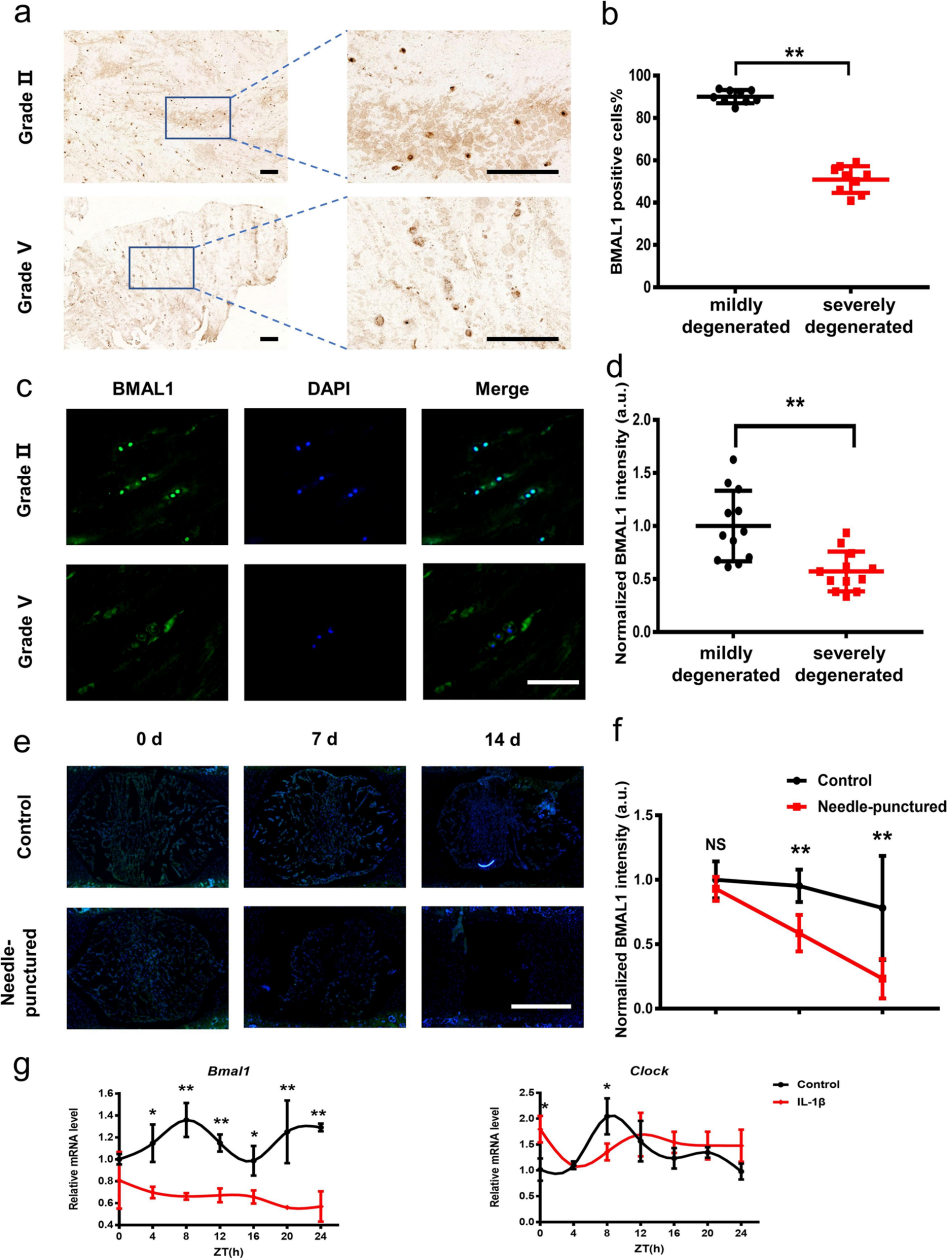
The BMAL1 expression of NP specimens in human donors, rat NP tissues, and cells. **a**, **b** Immunohistochemical staining images and quantification of BMAL1 in human NP tissues from the grade II and grade V groups. We selected 3 random fields in per individual for quantitative analysis (scale bar: 250 μm). *n* = 3, **P < 0.01. **c**, **d** Immunofluorescence staining images and quantification of BMAL1 in human NP tissues from the Grade II and Grade V groups. We selected 3 random fields in per individual for quantitative analysis (scale bar: 125 μm). *n* = 4, **P < 0.01. **e**, **f** Immunofluorescence staining images and quantification of BMAL1 in rat NP tissues from Control and puncture groups cultured 0 days, 7 days, and 14 days (scale bar: 500 μm). *n* = 3, NS, *P*>0.01, **P < 0.01. **g** The mRNA levels of *Bmal1* and *Clock* in control and IL-1β. *n* = 3, *P<0.05, **P < 0.01

Human NP tissues from 14 donors were divided into two groups (mildly group: grade II/III, severely group: grade IV/V) based on a modified Pfirrmann grading system [25]. HE staining was used to further confirm the accuracy of our grading process (Fig. S1a). IHC staining showed that the proportion of BMAL1-positive cells in the mildly group was significantly higher than that in the severely group (Fig. 1a, b). Consistent with IHC results, IF staining also showed a higher BMAL1 expression in mildly group (Fig. 1c, d).

HE and SO staining were used to evaluate the histological changes of rat IVDs in organotypic tissue-explant culture. A phenotype of disc degeneration was observed in the needle-punctured group, indicating a successful model building (Fig. S1b). IF staining of IVD sections revealed that the expression of BMAL1 was decreased in the needle-punctured group (Fig. 1e).

qRT-PCR showed a decreased anabolism (decreased expression of *Acan* gene) and an increased catabolism (increased expression of *Adamts5*, *Mmp3*, and *Mmp13* genes) in the IL-1β treated group when compared with the control group (Fig. S2a). Moreover, qRT-PCR also showed that the expression of *Bmal1* was decreased, and the rhythms of *Bmal1* and *Clock* were disturbed after 24 h of IL-1β treatment (Fig. 1f, g). The time phase and amplitudes of other clock genes, such as *Per1*, *Per2*, *Cry1*, and *Cry2*, were also disturbed after 24 h of IL-1β treatment (Fig. S2b). These results indicated that the disrupted IVD clock induced by inflammation might attribute to the process of IDD.

### Knockdown of Bmal1 led to the dampened peripheric clock and the disfunction of NP cells

**Fig. 2:**
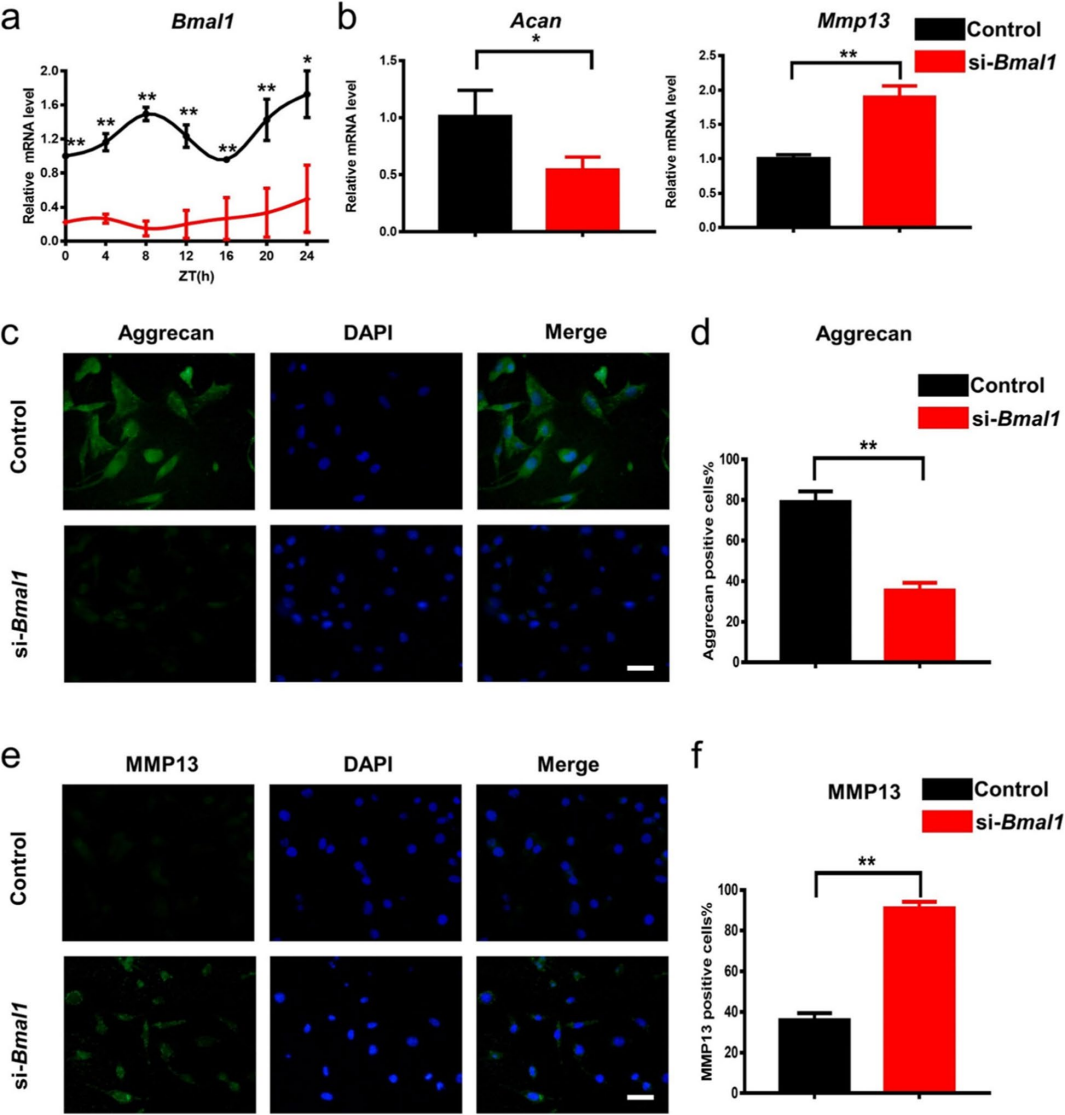
Knockdown of *Bmal1* led to the degeneration of NP cells in vitro. **a** The mRNA levels of *Bmal1* in the control and si-*Bmal1* groups. **b** The mRNA levels of *Acan* and *Mmp13* in the control and si-*Bmal1* groups. *n* = 3, *P<0.05, **P < 0.01. **c**–**f** Immunofluorescence staining images and quantification of Aggrecan and MMP13 in the control and si-*Bmal1* groups (scale bar: 25 μm). *n* = 3, **P < 0.01

To confirm the significant role of the peripheric clock in maintaining the homeostasis of NP, we used small interfering RNA (siRNA) to knock down the core clock gene *Bmal1* in NP cells. After transfection with siRNAs in NP cells, we screened out the most effective siRNA via qRT-PCR and Western blotting, which was shown in (Fig. S3a, b). Compared with the negative control group, the transcriptional level of *Bmal1* was significantly decreased, and the rhythm of it was obviously disturbed 24 h after the addition of si-*Bmal1*, which was verified by qRT-PCR in a time-proportioned sample process for 24 h (Fig. 2a). In addition, we observed a significantly decreased *Acan* mRNA expression level as well as an increased *Mmp13* mRNA expression level in the si-*Bmal1* group at 24 h after transfection (Fig. 2b, c). Consistent with qRT-PCR results, IF staining showed a lower Aggrecan expression and a higher MMP13 expression in the si-*Bmal1* group (Fig. 2d–g). These data indicated that the knockdown of *Bmal1* led to the degeneration of NP cells.

### Knockdown of Bmal1 decreased the expression of NRF2 and increased inflammatory response, oxidative stress reaction, and apoptosis of NP cells

**Fig. 3:**
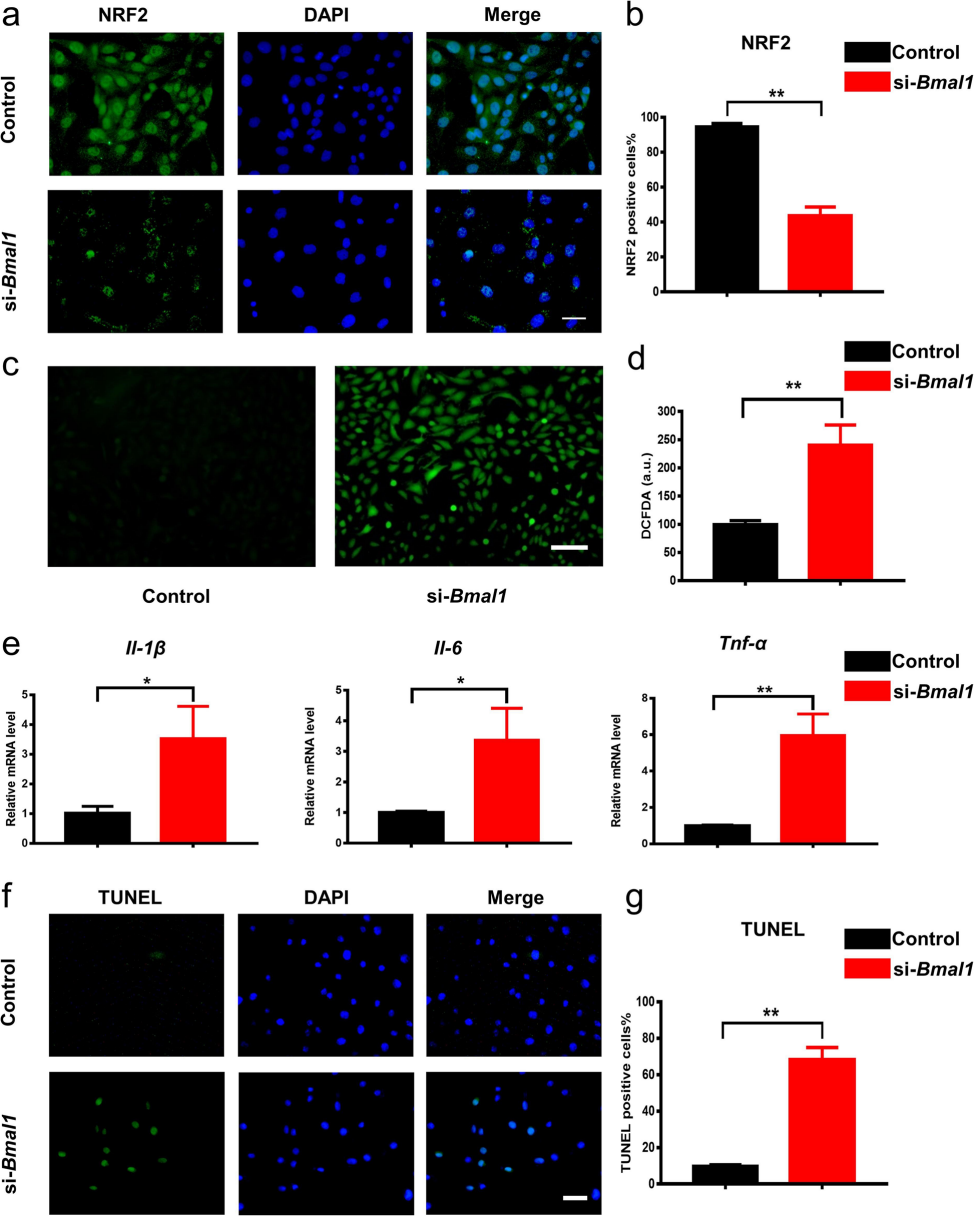
Si-*Bmal1* decreased the expression of NRF2 and increased the ROS, inflammatory response, and apoptosis. **a**, **b** Immunofluorescence staining images and quantification of NRF2 in the control and si-*Bmal1* groups (scale bar: 25 μm). *n* = 3, **P < 0.01. **c**, **d** DCFDA staining images and quantification in the control and si-*Bmal1* groups. *n* = 3, **P < 0.01. **e** The mRNA levels of *Il-1β*, *Tnf-α*, and *Il-6* in the control and si-*Bmal1* groups. *n* = 3, *P<0.05, **P < 0.01. **f**, **g** TUNEL staining and quantification in the control and si-*Bmal1* groups (scale bar: 25 μm). *n* = 3, **P < 0.01

It was reported that NRF2 expression and activity were under the control of the peripheric clock in lung, pancreatic tissue and macrophages [23, 24, 28]. In order to find the E-box site, a BMAL1 binding domain, in the *Nrf2* promoter, we confirmed that the E-box sequence (CAGCTG) existed in the promoter of *Nrf2* in rat NP cells by the JASPAR database, and the analysis of Find Individual Motif Occurrences (Fig. S4a, b). Next, we investigated whether the expression and activity of NRF2 in NP cells was under the control of the circadian clock. The results from qRT-PCR showed that a decreased expression of *Nrf2* dampened the circadian rhythm after the treatment of si-*Bmal1* (Fig. S4c). Consistent with qRT-PCR results, IF staining showed that the protein expression level of NRF2 was significantly decreased in the si-*Bmal1* treated group (Fig. 3a, b). These data showed that knockdown of *Bmal1* reduced the NRF2 expression and disturbed its rhythmic characteristics, indicating that *Bmal1* might play a major role in regulating the rhythmic expression of *Nrf2*. Moreover, we detected the ROS level and inflammatory response of NP cells after the knockdown of *Bmal1*. A significantly increased ROS level was observed in the si-*Bmal1* group (Fig. 3c, d). A significantly increased inflammatory cytokine expression, such as increased expression levels of *Il-1β, Tnf-α,* and *Il-6*, was also observed in the si-*Bmal1* group (Fig. 3e).

Next, we detected the apoptosis of NP cells caused by *Bmal1* knockdown via TUNEL staining. The results showed that knockdown of *Bmal1* increased the apoptosis of NP cells (Fig. 3f, g). Given that *Nrf2* is under the transcriptional control of BMAL1, we speculated that knockdown of *Bmal1* leads to increased inflammatory response, oxidative stress reaction, and apoptosis of NP cells by reducing the expression of NRF2.

### NRF2 activation attenuated the dysfunction of NP cells induced by the dampened IVD clock

**Fig. 4:**
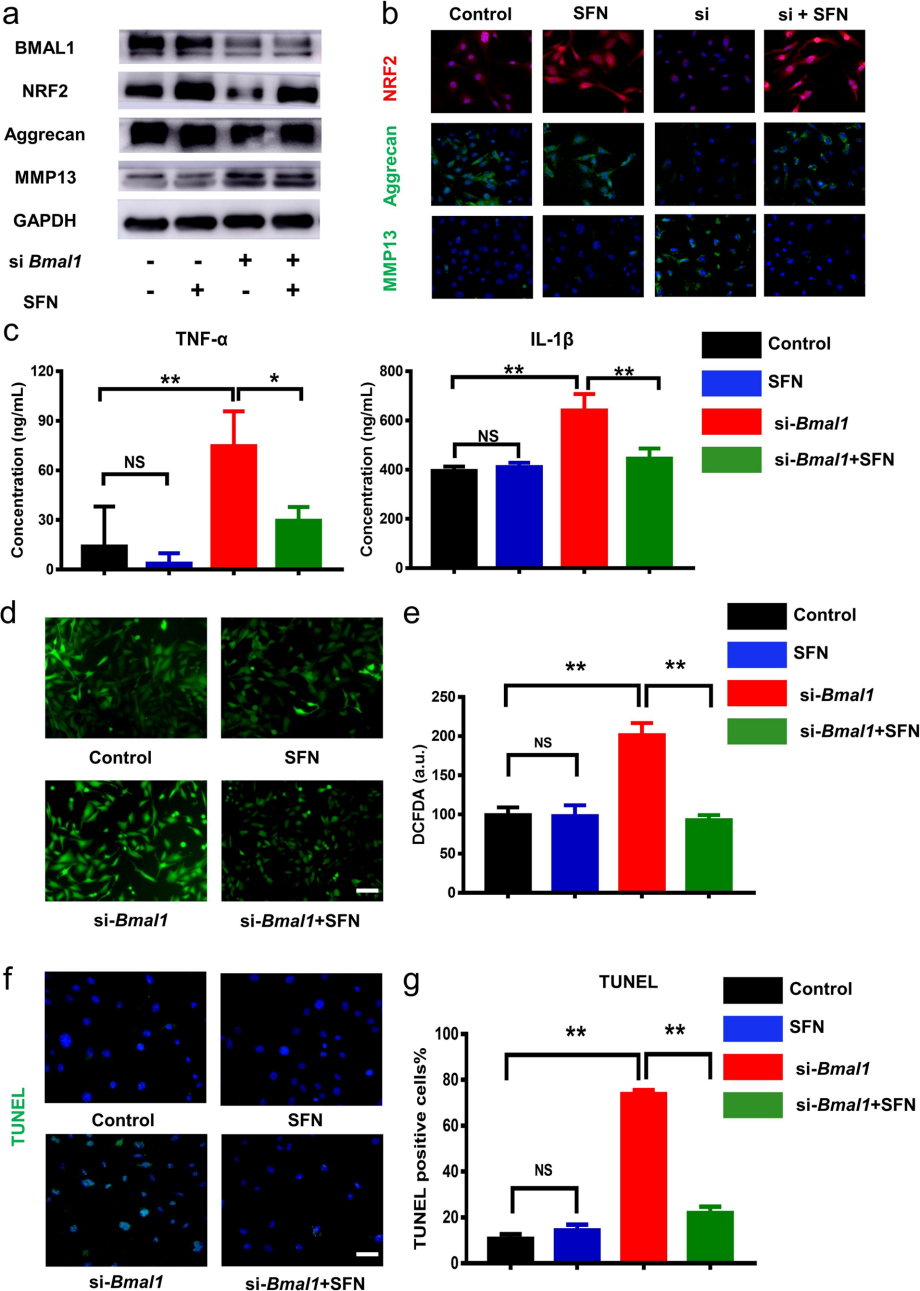
SFN attenuated si-*Bmal1*-induced the dysfunction of NP cells. The NP cells were divided into four groups, with no treatment, incubated with 5 μM SFN, si-*Bmal1*, and si-*Bmal1* + 5 μM SFN. **a** Western blotting of BMAL1, NRF2, Aggrecan, and MMP13 protein expression (normalized to GAPDH expression). **b** Immunofluorescence staining of NRF2, Aggrecan, and MMP13 staining in Control, SFN, si-*Bmal1*, and si-*Bmal1* + 5 μM SFN group (scale bar: 25 μm). **c** The levels of TNF-α and IL-1β protein. n = 3, NS, not significant difference, *P<0.05, **P < 0.01. **d**, **e** DCFDA staining images and quantification (scale bar: 50 μm). **f**, **g** TUNEL staining images and quantification (scale bar: 25 μm)

To confirm whether the dampened clock-controlled gene *Nrf2* is one major cause of IDD, we examined the NRF2 expression level in human NP tissues. IF results showed that the proportion of NRF2-positive cells was significantly higher in the mildly group (Grade II/III), compared with the severely group (Grade IV/V) (Fig. S5a, b). Next, we used SFN, a NRF2 agonist, to rescue the phenotypes of IDD induced by the dampened IVD clock. After transfection with si-*Bmal1* or treated with SFN for 48 h, the Western blotting analysis showed no change of BMAL1 in the SFN group and significant decreases of BMAL1 in both si-*Bmal1* group and si-*Bmal1* + SFN group at the protein level (Fig. 4a). However, a significant increase of NRF2 was observed in both the SFN group and the si-*Bmal1* + SFN group, while a significant decrease of NRF2 was observed only in the si-*Bmal1* group (Fig. 4a). These results indicated that *Nrf2* was the downstream target of the core clock protein BMAL1. Moreover, compared with the si-*Bmal1* group, a higher level of anabolic marker Aggrecan and a lower level of catabolic marker MMP13 were observed in the si-*Bmal1* + SFN group (Fig. 4a). Consistent with Western blotting results, IF assays showed the same results (Fig. 4b). These results indicated that activation of clock-controlled gene *Nrf2* was effective enough to recover the disturbed metabolism induced by the dampened IVD clock. Next, we explored the influence of SFN on the inflammatory response, oxidative stress reaction and apoptosis of NP cells induced by si-*Bmal1*. Compared with the si-*Bmal1* group, we observed significantly decreased transcriptional levels of *Il-1β*, *Tnf-α*, and *Il-6* in the si-*Bmal1* + SFN group (Fig. S5c). ELISA results also further confirmed the results from qRT-PCR (Fig. 4c). The increased ROS level was observed in si-*Bmal1* group, while the addition of SFN significantly reduced the ROS level induced by si-*Bmal1* (Fig. 4d, e). In addition, SFN treatment also significantly reduced the TUNEL-positive cells induced by si-*Bmal1*, indicating its strong anti-apoptotic ability (Fig. 4f, g). These results showed that targeting the clock-controlled gene *Nrf2* was an effective treatment to ameliorate the phenotypes of IDD induced by the dampened IVD clock.

### SFN attenuated the degenerative process of NP tissues in an organotypic tissue-explant model

**Fig. 5:**
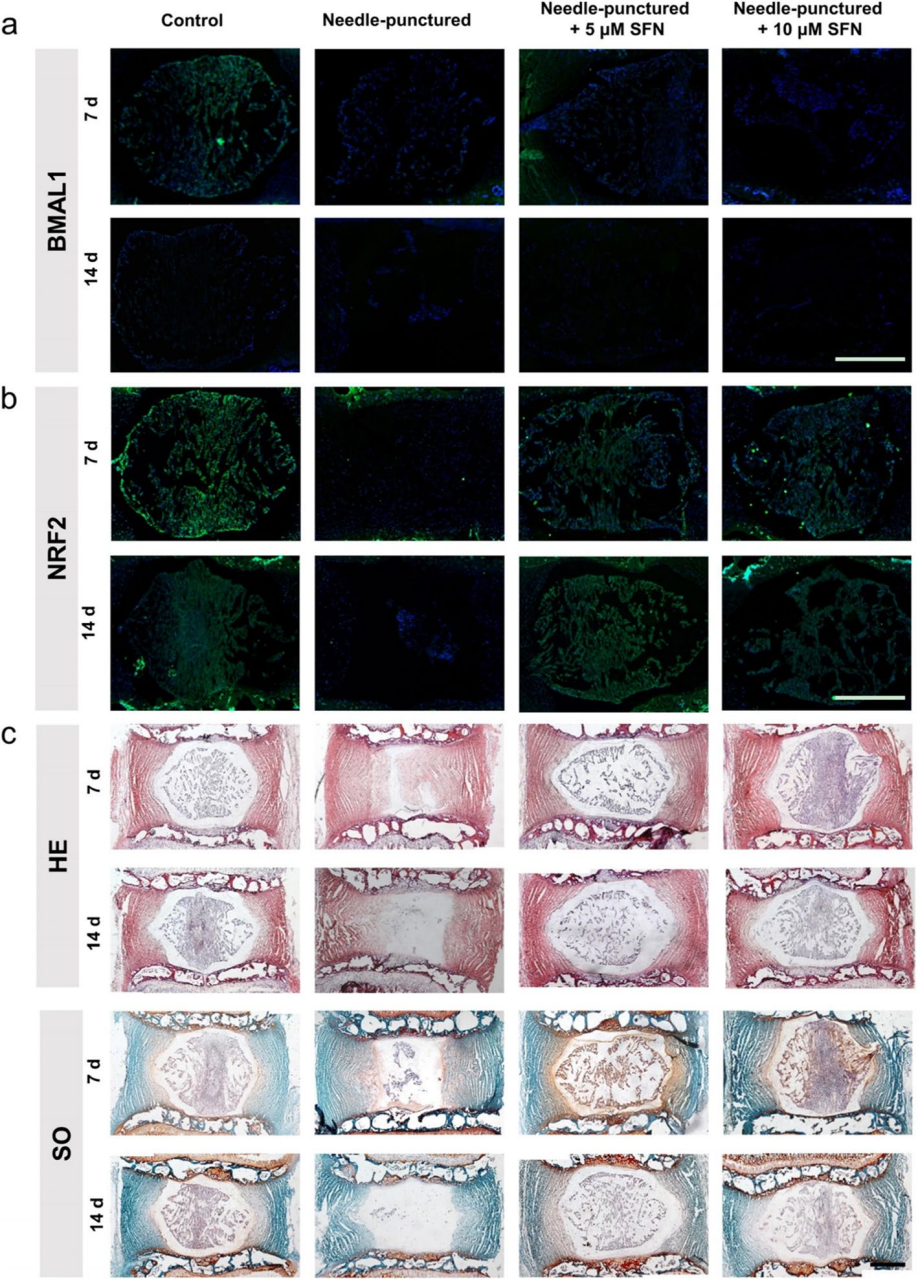
SFN attenuated the degeneration of rat NP tissues in an organotypic tissue-explant model. The NP tissues were divided into four groups, control, needle-punctured, needle-punctured + 5 μM SFN, and needle-punctured + 10 μM SFN. **a** Immunofluorescence staining of BMAL1 in rat NP tissues cultured 7 days and 14 days (scale bar: 500 μm). **b** Immunofluorescence staining of NRF2 in rat NP tissues cultured for 7 days and 14 days (scale bar: 500 μm). **c** HE and safranin O staining of the rat IVDs cultured for 7 days and 14 days (scale bar: 500 μm)

Sulforaphane (SFN) is an isothiocyanate compound. Glucoraphanin, the precursor form of SFN, is widely found in cruciferous plants [29]. In vivo, glucoraphanin can be hydrolyzed by microorganisms in the intestine to produce active SFN, which is then absorbed by the body. SFN enters the systemic circulation to play biological functions, such as anti-tumor, anti-oxidative damage, anti-bacteria, and immune regulation [27]. Recent studies have shown that SFN can alleviate progerin-induced IDD [28]. Next, we explored the therapeutic effect of SFN in an organotypic tissue-explant model. IF staining showed significantly decreased expressions of BMAL1 and NRF2 in the needle-punctured group when compared with the control group. Addition of SFN significantly increased the expression of NRF2 in this punctured model, while the expression of BMAL1 showed no obvious changes (Fig. 5a, b). The results from HE and SO staining further confirmed the therapeutic effect of SFN (Fig. 5c). These results indicated that targeting the clock-controlled gene *Nrf2* was an effective method for ameliorating inflammation-induced IDD.

## Discussion

Peripheral rhythm has been proved essential for maintaining tissue-specific functions, and IVDs have been confirmed a rhythmic organ [10]. Recently, some degenerative characteristics have been observed in BMAL1-deficiency mouse IVDs [19], indicating that the peripheral clock plays an important role in maintaining the homeostasis of IVD tissues. However, the pathogenesis of IDD induced by the dampened molecular clock is still unclear. Here, we confirmed that the clock-controlled gene *Nrf2* was the direct target of the core clock molecule BMAL1 in IVDs, and provided a potential intervention target to prevent the process of IDD caused by the dampened periphery clock.

NRF2, a transcription factor containing the basic structure of the leucine zipper, showed an important role in regulating inflammation and redox reaction [30]. Lee et al. reported that the expression of *Nrf2* was under the direct control of BMAL1 in pancreatic tissues by binding to the E-box of the *Nrf2* promoter region [24]. Another study also showed a clock-controlled expression of *Nrf2* in mouse macrophages by binding to the E-box sequence of the *Nrf2* promoter [28]. Our study for the first time demonstrated that the rhythmic expression of *Bmal1* and *Nrf2* were dampened after knockdown of the *Bmal1* gene in nucleus pulposus cells, while upregulating the downstream NRF2 expression successfully ameliorated some phenotypes of IDD induced by the disruption of IVD clock.

There are still some limitations of this study. Although SFN, a kind of isothiocyanate widely existing in broccoli, water, cabbage, and other cruciferous plants, has been reported to protect against oxidative stress and inflammatory responses by activating the Nrf2/ARE pathway in a variety of cells, some non-specific biological functions of it have not been total excluded [31, 32]. In addition, because of the limitation of sample size, the results of human NP tissue samples can only provide preliminary clues for our study. Further investigations with a large sample size and the use of appropriate knock-out mice will help solve these problems.

In a word, this study offers an opinion on the mechanism of NP disfunction induced by the dampened peripheric clock. The loss of BMAL1 in NP cells results in the decreased expression of its downstream target gene *Nrf2*, which thus leads to the increased expression of intracellular proinflammatory factors, enhanced oxidative stress, increased apoptosis, and degradation of ECM. SFN successfully ameliorates the IDD phenotypes induced by decreased BMAL1 via increasing the expression of NRF2 and reducing the inflammatory response and oxidative stress.

## Conclusions

This study extends the relationship between peripheral clock and IVD homeostasis and provides a potential therapeutic method for the prevention and recovery of IDD by targeting the clock-controlled gene *Nrf2*.

## Supplementary Information

**Additional file 1: Figure S1.** Histochemical stainning of NP specimens in donors and rat NP tissues. (a) HE staining of the human NP tissues from the Grade II, Grade III, Grade IV and Grade V groups (scale bar: 250 μm). (b) HE and SO staining of rat NP tissues from Control and puncture groups cultured 0 day, 7 days and 14 days (scale bar: 500 μm). **Figure S2.** The expression of anabolic or catabolic genes of intervertebral disc and clock genes in rat NP cells. (a) The mRNA levels of *Acan*, *Adamts5*, *Mmp3* and *Mmp13* in Control and IL-1β group. (b) The mRNA levels of *Per1*, *Per2*, *Cry1* and *Cry2* in Control and IL-1β group. **Figure S3.** The efficiencies of knock down *Bmal1* of NP cells by different si RNAs. (a) The efficiencies of knock down *Bmal1* of NP cells by si RNAs were determined by qRT-PCR. n = 3, NS, not significant difference, *P<0.05, **P < 0.01. (b) The efficiencies of knock down *Bmal1* of NP cells by si RNAs were determined by western blot. **Figure S4.** BMAL1 regulates *Nrf2* by combining with E-box. (a) The motif of E-box. (b) The situation of E-box sequence (CAGCTG) existed in the promoter of *Nrf2*. (c) The mRNA levels of *Nrf2* in Control and si-*Bmal1* group. **Figure S5.** The NRF2 expression of NP specimens in donors and rat NP cells. (a) Immunofluorescence staining of NRF2 in human NP tissues from the Grade II and Grade V groups (scale bar: 125 μm). (b) Quantification of NRF2 expression in human NP tissues from the Grade II/III and Grade IV/V groups. n = 12, **P < 0.01. (c) *Nrf2*, *Il-1β*, *Tnf-α* and *Il-6* mRNA levels of Control, SFN, si-*Bmal1* and si-*Bmal1*+5 μM SFN groups were determined by qRT-PCR. n = 3, NS, not significant difference, *P<0.05, **P < 0.01. **Supplementary Table 1.** The sequences of siRNA targeting *Bmal1.* **Supplementary Table 2.** The primers used for qRT-PCR. **Supplementary Table 3.** Histological grading scale of intervertebral disc.

## Data Availability

All original data supporting the conclusions are provided by the authors.

## Abbreviations

IVD: Intervertebral disc
IDD: Intervertebral disc degeneration
NP: Nucleus pulposus
ECM: Extracellular matrix
CCGs: Clock-controlled genes
SFN: Sulforaphane
HE: Hematoxylin-eosin
SO: Safranin O-fast green
IHC: Immunohistochemistry
IF: Immunofluorescence
DAPI: 4′,6-diamidino-2-phenylindole
ROS: Reactive oxygen species
DCFDA: 2′,7′-dichlorofluorescein diacetate
