# Supplementary material for: Targeting clock-controlled gene Nrf2 ameliorates inflammation-induced intervertebral disc degeneration
Source: Arthritis Res Ther. 2022 Aug 3;24:181. doi: 10.1186/s13075-022-02876-w (PMC9347076; doi:10.1186/s13075-022-02876-w)
Supplement: Supplementary file 1 — Additional file 1: Figure S1. Histochemical stainning of NP specimens in donors and rat NP tissues. (a) HE staining of the human NP tissues from the Grade II, Grade III, Grade IV and Grade V groups (scale bar: 250 μm). (b) HE and SO staining of rat NP tissues from Control and puncture groups cultured 0 day, 7 days and 14 days (scale bar: 500 μm). Figure S2. The expression of anabolic or catabolic genes of intervertebral disc and clock genes in rat NP cells. (a) The mRNA levels of Acan, Adamts5, Mmp3 and Mmp13 in Control and IL-1β group. (b) The mRNA levels of Per1, Per2, Cry1 and Cry2 in Control and IL-1β group. Figure S3. The efficiencies of knock down Bmal1 of NP cells by different si RNAs. (a) The efficiencies of knock down Bmal1 of NP cells by si RNAs were determined by qRT-PCR. n = 3, NS, not significant difference, *P<0.05, **P < 0.01. (b) The efficiencies of knock down Bmal1 of NP cells by si RNAs were determined by western blot. Figure S4. BMAL1 regulates Nrf2 by combining with E-box. (a) The motif of E-box. (b) The situation of E-box sequence (CAGCTG) existed in the promoter of Nrf2. (c) The mRNA levels of Nrf2 in Control and si-Bmal1 group. Figure S5. The NRF2 expression of NP specimens in donors and rat NP cells. (a) Immunofluorescence staining of NRF2 in human NP tissues from the Grade II and Grade V groups (scale bar: 125 μm). (b) Quantification of NRF2 expression in human NP tissues from the Grade II/III and Grade IV/V groups. n = 12, **P < 0.01. (c) Nrf2, Il-1β, Tnf-α and Il-6 mRNA levels of Control, SFN, si-Bmal1 and si-Bmal1+5 μM SFN groups were determined by qRT-PCR. n = 3, NS, not significant difference, *P<0.05, **P < 0.01. Supplementary Table 1. The sequences of siRNA targeting Bmal1. Supplementary Table 2. The primers used for qRT-PCR. Supplementary Table 3. Histological grading scale of intervertebral disc. [file 13075_2022_2876_MOESM1_ESM.docx]

**Supplementary Materials**


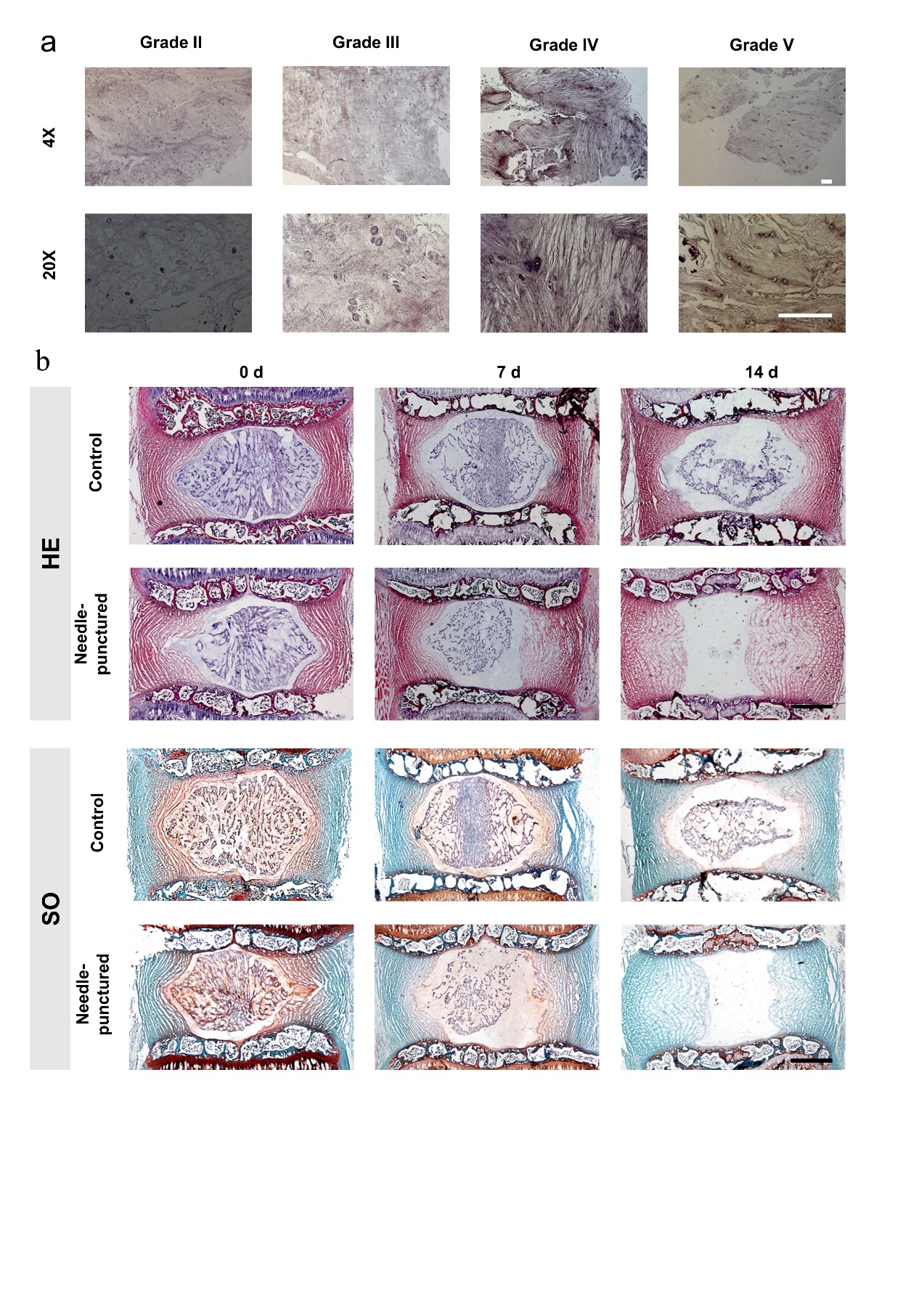


**Figure S1. Histochemical stainning of NP specimens in donors and rat NP tissues.** (a) HE staining of the human NP tissues from the Grade II, Grade III, Grade IV and Grade V groups (scale bar: 250 μm). (b) HE and SO staining of rat NP tissues from Control and puncture groups cultured 0 day, 7 days and 14 days (scale bar: 500 μm).


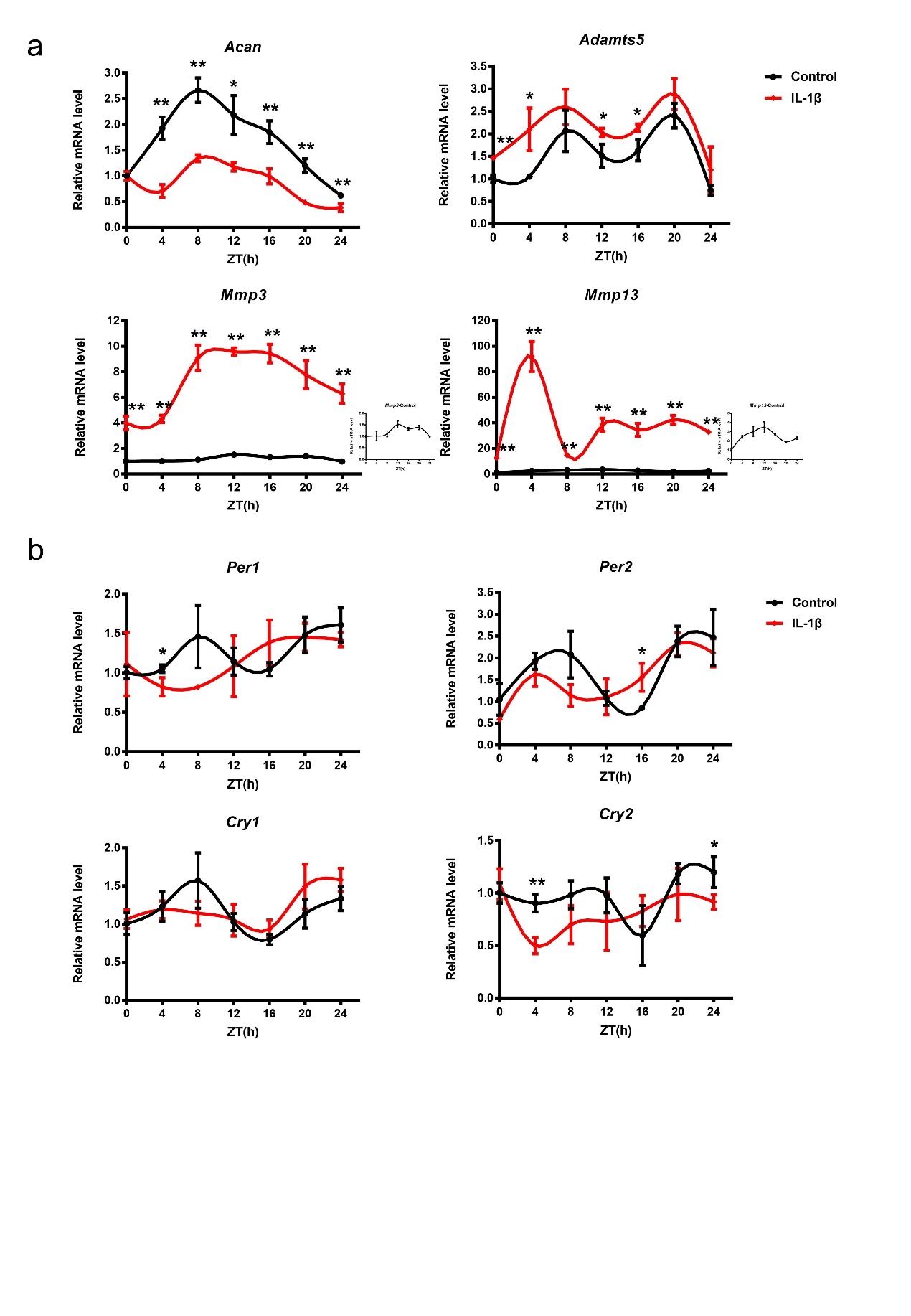


**Figure S2. The expression of anabolic or catabolic genes of intervertebral disc and clock genes in rat NP cells.** (a) The mRNA levels of *Acan*, *Adamts5*, *Mmp3* and *Mmp13* in Control and IL-1β group. (b) The mRNA levels of *Per1*, *Per2*, *Cry1* and *Cry2* in Control and IL-1β group.


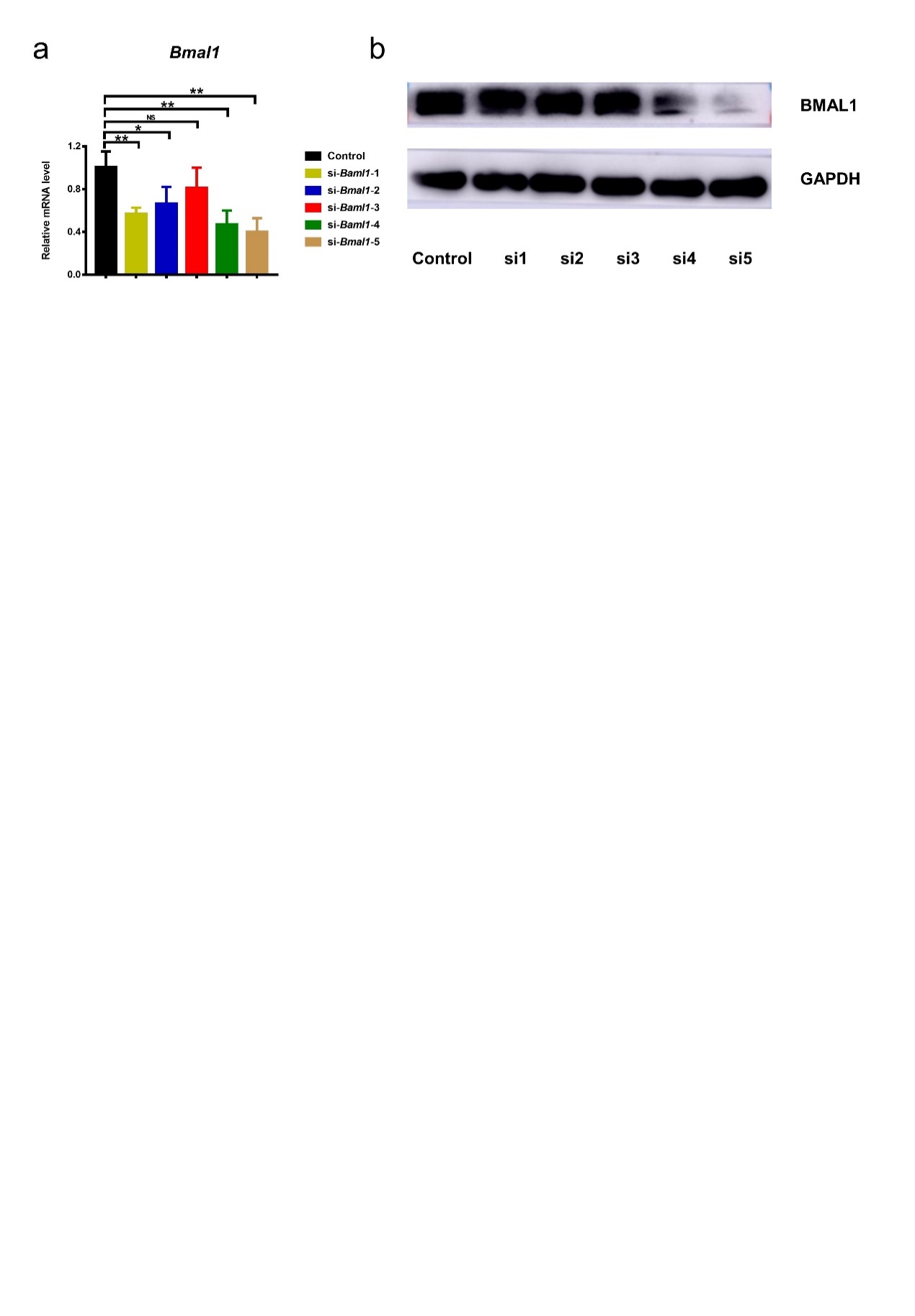


**Figure S3. The efficiencies of knock down *Bmal1* of NP cells by different si RNAs.** (a) The efficiencies of knock down *Bmal1* of NP cells by si RNAs were determined by qRT-PCR. n = 3, NS, not significant difference, *P＜0.05, **P < 0.01. (b) The efficiencies of knock down *Bmal1* of NP cells by si RNAs were determined by western blot.


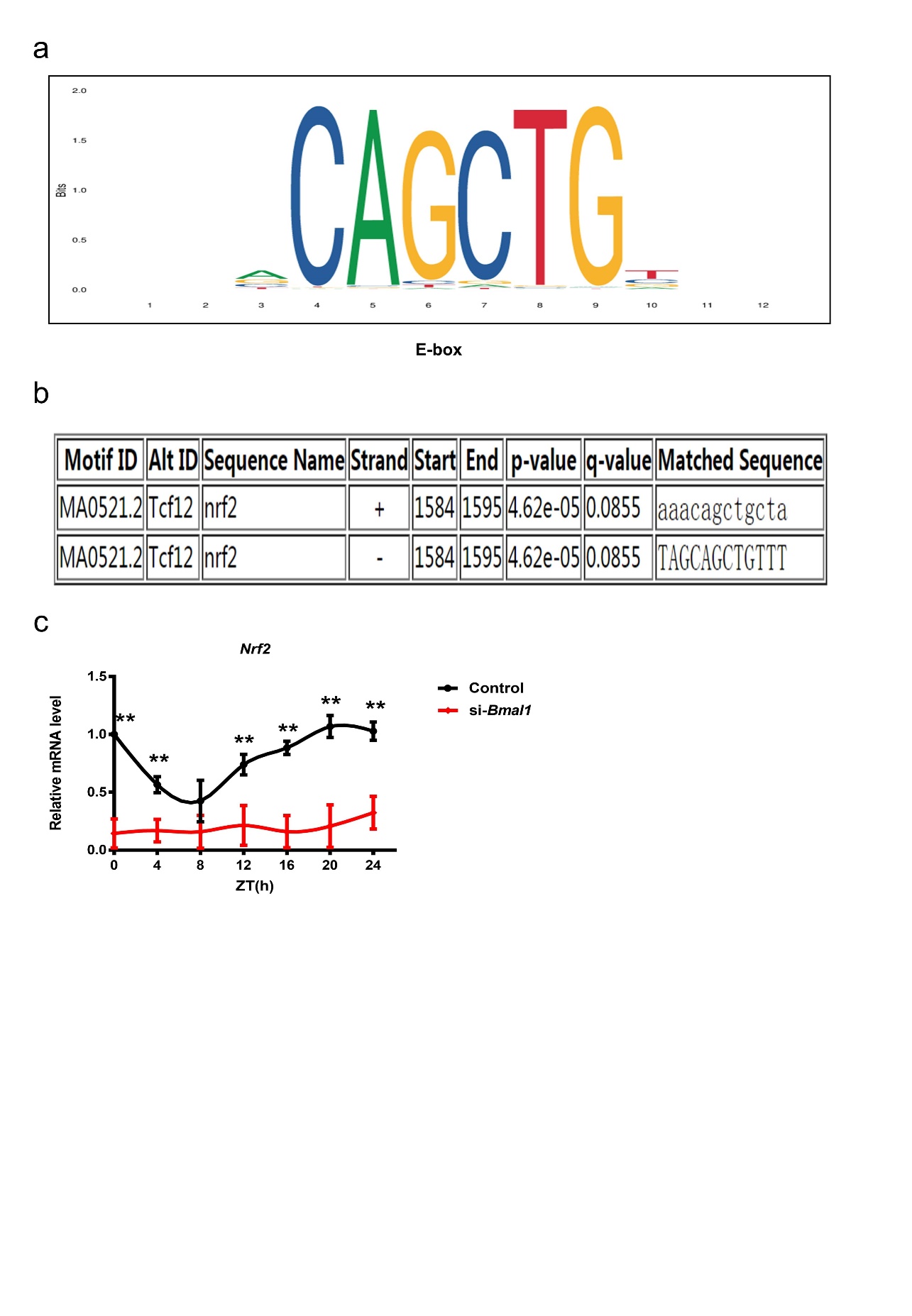


**Figure S4. BMAL1 regulates *Nrf2* by combining with E-box.** (a) The motif of E-box. (b) The situation of E-box sequence (CAGCTG) existed in the promoter of *Nrf2*. (c) The mRNA levels of *Nrf2* in Control and si-*Bmal1* group.


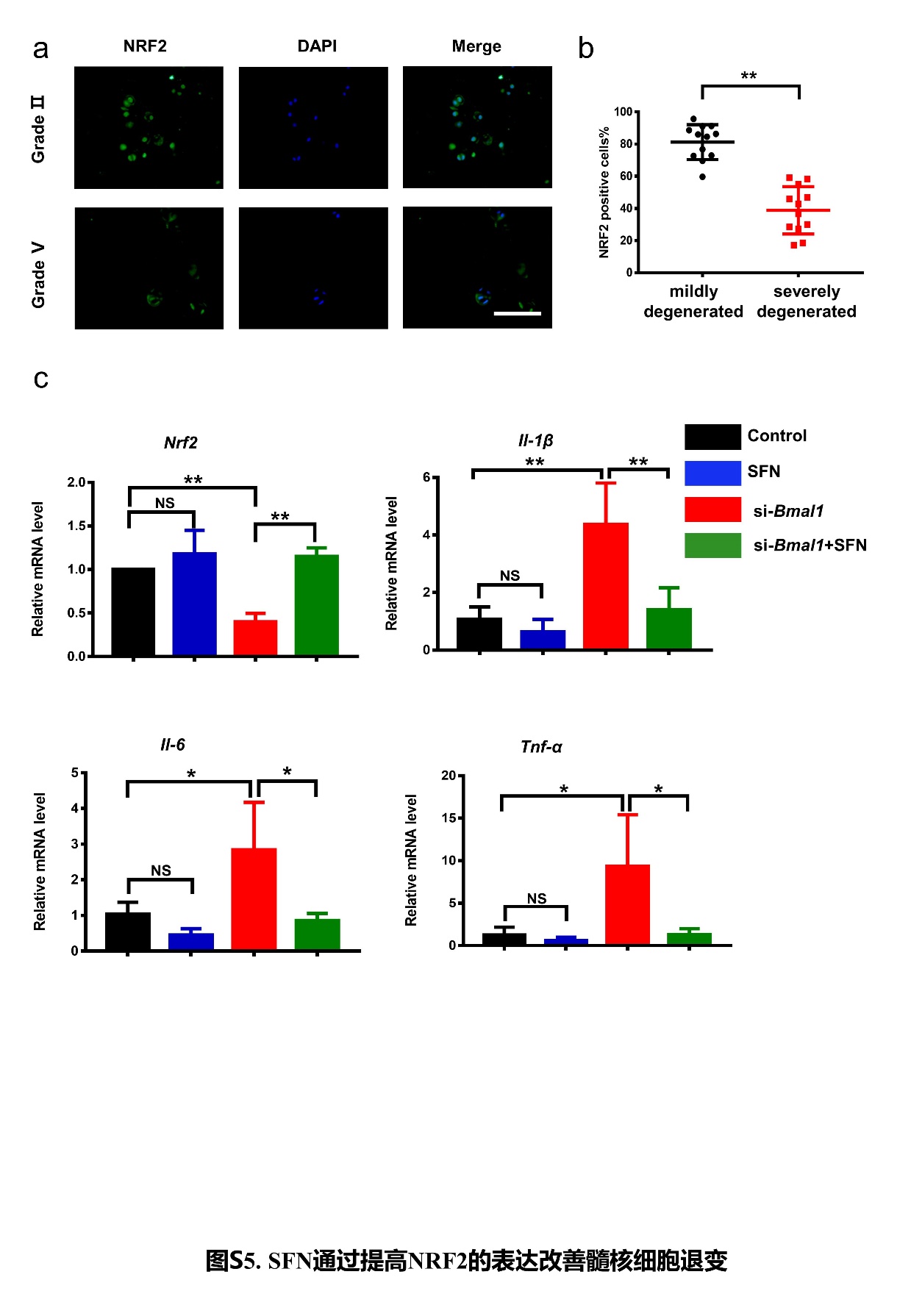


**Figure S5. The NRF2 expression of NP specimens in donors and rat NP cells.** (a) Immunofluorescence staining of NRF2 in human NP tissues from the Grade II and Grade V groups (scale bar: 125 μm). (b) Quantification of NRF2 expression in human NP tissues from the Grade II/III and Grade IV/V groups. n = 12, **P < 0.01. (c) *Nrf2*, *Il-1β*, *Tnf-α* and *Il-6* mRNA levels of Control, SFN, si-*Bmal1* and si-*Bmal1*+5 μM SFN groups were determined by qRT-PCR. n = 3, NS, not significant difference, *P＜0.05, **P < 0.01.

**Supplementary Table 1. The sequences of siRNA targeting *Bmal1***

| Gene(Rattus) | Sequence(5’- 3’) |
| --- | --- |
| si1 | sense GCCAACAUUUCUAUCCGAUTT |
|  | antisense AUCGGAUAGAAAUGUUGGCTT |
| si2 | sense CCGAGGGAAGAUCCUCUUUTT |
|  | antisense AAAGAGGAUCUUCCCUCGGTT |
| si3 | sense CCUCAAUUAUAGCCAGAAUTT |
|  | antisense AUUCUGGCUAUAAUUGAGGTT |
| si4 | sense GCAACAGGCCUUCAGUAAATT |
|  | antisense UUUACUGAAGGCCUGUUGCTT |
| si5A | sense CCAAGGAAGUAGAAUACAUTT |
|  | antisense AUGUAUUCUACUUCCUUGGTT |
| si5B | sense GCAUCGACAUGAUAGACAATT |
|  | antisense UUGUCUAUCAUGUCGAUGCTT |
| si5C | sense CAACAGCUACAGUAUCAAATT |
|  | antisense UUUGAUACUGUAGCUGUUGTT |

**Supplementary Table 2. The primers used for qRT-PCR**

| Gene(Rattus) | Sequence(5’- 3’) |
| --- | --- |
| *Bmal1* | Forward primer GACTTCGCCTCCACCTGTTCAA |
|  | Reverse primer GCAGCCCTCATTGTCTGGTTCA |
| *Nrf2* | Forward primer TTGTAGATGACCATGAGTCGC |
|  | Reverse primer ACTTCCAGGGGCACTGTCTA |
| *Acan* | Forward primer AGGATGGCTTCCACCAGTGC |
|  | Reverse primer TGCGTAAAAGACCTCACCCTCC |
| *Mmp13* | Forward primer GCAGCTCCAAAGGCTACAA |
|  | Reverse primer CATCATCTGGGAGCATGAAA |
| *Il-1β* | Forward primer GACTTCACCATGGAACCCGT |
|  | Reverse primer GGAGACTGCCCATTCTCGAC |
| *Il-6* | Forward primer CCCAACTTCCAATGCTCTCCT |
|  | Reverse primer TAGCACACTAGGTTTGCCGAG |
| *Tnf-α* | Forward primer GGCGTGTTCATCCGTTCTCT |
|  | Reverse primer CCCAGAGCCACAATTCCCTT |
| *Clock* | Forward primer TCAACTCAGAGTCAACAGAGTGT |
|  | Reverse primer ACTCTGGGTGCTATTCTGCG |
| *Per1* | Forward primer GTGCATCTCAGCGGAGTTCT |
|  | Reverse primer CACTGGTAGACGGGTTGTCC |
| *Per2* | Forward primer CTGCGAAGCGCCTCATTCC |
|  | Reverse primer TTATGCTCCGCCTCTGTCATC |
| *Cry1* | Forward primer GCGGAAACTGCTCTCAAGGA |
|  | Reverse primer CCCGCATGCTTTCGTATCAG |
| *Cry2* | Forward primer CCGATGGAGGTTCCTACTGC |
|  | Reverse primer TGCATCCCGTTCTTTCCCAA |
| *Gapdh* | Forward primer AGTGCCAGCCTCGTCTCATA |
|  | Reverse primer GACTGTGCCGTTGAACTTGC |

**Supplementary Table 3. Histological grading scale of intervertebral disc**

**Histological grading scale of intervertebral disc (IVD)**

**Nucleus pulposus (NP)**

5. Large, bulging central cavity with abundant NP material; more than 2/3 IVD height; smooth borders with minimal disruption

4. Slightly reduced central cavity size with some NP material present; more than 1/3 IVD height and less than 2/3 IVD height; minimal border disruption may be present

3. Markedly reduced and disrupted cavity with minimal NP material and compartmentalization; total cavity; more than 1/3 IVD height and less than 2/3 IVD height

2. Severe disruption of NP with minimal cavity; total cavity less than 1/3 IVD height but more than 0; consists only of a few small pockets lined by NP-like cells

1. Complete obliteration of cavity with no NP-lined pockets

**Annulus fibrosus (AF)**

5. Discrete, well-opposed lamellae bulging outward with no infolding; minimal preparation defect with ‘‘simple radial clefting’’

4. Discrete lamellae, less well-opposed; minimal infolding may be present; fibers remain well-organized, but with ‘‘complex radial clefting’’

3. Moderate to severe infolding of discrete, relatively well-opposed lamellae; moderate fragmentation of lamellae; AF fibers remain well organized

2. Severe infolding and distortion of poorly opposed lamellae; severe fragmentation of lamellae; small regions of disorganized fibrous material replacing central lamellae

1. Severe infolding, distortion, and fragmentation of lamellae; extensive amount of disorganized fibrous material replacing central lamellae

**Safranin O-fast green (SO) staining**

5. Deep red stain in peri-NP region; deep red stain between AF lamellae with gradual fading in periphery

4. Pattern of stain similar to grade 5, but with less prominent peri-NP stain and more rapid fading of stain between AF lamellae

3. Normal pattern no longer present; large areas of faded red centrally and in AF, but inconsistent and patchy

2. Only 1-3 small patches of faded red stain either centrally or in AF

1. Entire IVD completely washed out with no red stain
